# The Late Dr. Steele

**Published:** 1893-08-05

**Authors:** 


### THE LATE DR. STEELE.

In the chapel of Guy's Hospital is now erected an appropriate and beautiful memorial tablet to the late lamented Superintendent of the Hospital. The tablet is of fine coloured marble. A border of mosaic encloses the inscription, which runs thus:—

In Memory
of
John Charles Steele, M.D.,
Who died at his post after forty years' service as
Superintendent of Guy's Hospital,
November 6th, 1892,
In the 71st year of his age.
The kindness of heart and large experience which
Enabled him to perform so faithfully
The manifold duties of his office,
Will be long remembered
By the Governors and Staff
Who have caused this tablet to be erected.

